# Determination of Phenolic Compounds and Bioactive Potential of Plum (*Prunus salicina*) Peel Extract Obtained by Ultrasound-Assisted Extraction

**DOI:** 10.1155/2022/7787958

**Published:** 2022-08-02

**Authors:** Muhammad Jawad, Moazzam Ali, Sadia Qasim, Ali Akbar, Nazir Ahmad Khan, Muhammad Bilal Sadiq

**Affiliations:** ^1^School of Life Sciences, Forman Christian College (A Chartered University), Lahore 54600, Pakistan; ^2^School of Biochemistry and Biotechnology, University of the Punjab, Lahore, Pakistan; ^3^The University of Child Health and Children's Hospital, Lahore, Pakistan; ^4^Department of Microbiology, University of Balochistan, Pakistan; ^5^Department of Animal Nutrition, The University of Agriculture Peshawar, Pakistan

## Abstract

Ultrasound-assisted extraction (UAE) of bioactive compounds from black plum peels was optimized by response surface methodology (RSM). Temperature (35-55°C), time (15-45 min), and ethanol concentration (50-90%) were selected as independent extraction parameters, whereas total anthocyanin content (TAC), total phenolic content (TPC), and 2,2-diphenyl-1-picrylhydrazyl (DPPH) inhibition were kept as response variables. The optimized extraction conditions were determined by RSM as extraction at 49°C for 37 min with 68% ethanol, which corresponded to TAC, TPC, and DPPH inhibition values of 5.42 ± 0.61 mg/g, 6.217 ± 0.76 mg GAE/g, and 89 ± 2.13%, respectively. Fourier-transform infrared spectrometer (FTIR), high-performance liquid chromatography (HPLC), and gas chromatography mass spectrometer (GCMS) were used for chemical characterization of optimized plum peel extract (PPE). Optimized PPE was further evaluated for antibacterial, antioxidant, anticancer, and food preservation potential. PPE showed 92.31% DPPH inhibition with IC_50_ value of 360.6 *μ*g/ml. Optimized PPE extract was effective in the inhibition of cancer cell proliferation and migration, and IC_50_ values were in the range 1.85-3.96 mg/ml for different human cancer cell lines. Major phenolics identified in PPE were ferulic acid (47.87 mg/kg), sinapic acid (9.15 mg/kg), quercetin (7.44 mg/kg), gallic acid (3.24 mg/kg), m-coumaric acid (2.59 mg/kg), and vanillic acid (1.12 mg/kg). PPE extract inhibited the growth of various foodborne bacterial pathogens and increased the shelf life of PPE coated fresh grapes. PPE due to antibacterial, anticancer, antioxidant, and food preservation potential can be used in developing functional food and pharmaceutical products.

## 1. Introduction

Plum (*Prunus salicina*) is a popular fruit and shares the family Rosaceae with peach and apricot. Plum contains a broad variety of flavors (from sour to sweet) and colors (black, yellow, red, and purple) and its peels are comprised of approximately 10-20% of the total weight of plum fruit [1]. Plums are processed either by freezing, canning, juicing, or drying [2]. However, drying is the oldest and most common way of plum processing [3]. A significant amount of plum peel remains as a byproduct after drying and processing of plum, and due to the presence of bioactive compounds, plum peels can be used to develop nutraceuticals and functional food products [4, 5].

Phenolic compounds are well-known bioactive molecules for their health promoting ability and effectiveness against various diseases [6]. Black plum peel (BPP) has wide variety of bioactive compounds such as phenolic acids, anthocyanins, carotenoids, flavanols, and various other aromatic compounds [7, 8]. Plums have a special attraction due to their color, taste, and nutritional value. Anthocyanins are a type of naturally occurring phenolic chemicals that give fruits their color [9]. Most of the anthocyanins are present in plum peel [10], which are responsible for their deep color [11]. Anthocyanins are known for their high antioxidant potential which makes them vital for human health. [12]. Plum peels are rich source of anthocyanins and various phenolic compounds (ferulic acid, chlorogenic acid, kaempferol, and p-hydroxybenzoic acid) which are associated with various health benefits such as antioxidant, anticancer, and antimicrobial potential [13, 14].

Free radicals are produced during metabolism, and their excessive production can result in multiple diseases of brain and bones [15]. Antioxidants protect cells by free radical scavenging and convert them into nonradical species [16]. Bioactive compounds from plant sources can be used as natural antioxidants due to their free radical scavenging capacity and safety in comparison to synthetic antioxidants [17]. Due to high content of bioactive compounds, plum fruit exhibits strong antioxidant, anticancer, antihyperglycemic, antihypertensive, and laxative properties, and its consumption has been linked to better cognitive function and bone health [10, 18]. Food preservation systems such as edible coatings can be developed based on natural preservatives from plants in combination with other edible polymers. Chitosan is nontoxic polysaccharide with good film forming capacity and can be used to develop biodegradable edible coatings [19].

Due to sensitive nature, extraction of bioactive compound from plant sources has always been a major concern. Conventional extraction techniques are time and solvent intensive and require a substantial amount of energy [20]. Ultrasonic-assisted extraction (UAE) is an innovative, green, and rapidly evolving technology that has a significant advantage over conventional extraction methods, including better extraction yield, less time, and solvent consumption [21, 22]. Due to variety of health benefits associated with fruit peels, the bioactive compounds extracted from peels can be used in the formulation of food preservation systems, nutraceutical, pharmaceutical, and medicinal products. In this study, UAE was used to extract bioactive compounds from plum peels and evaluated for their antioxidant, antimicrobial, and anticancer potential.

## 2. Materials and Methods

Black plum (*Prunus Salicina* L.) fruit samples were purchased from the local market of Sialkot city of Pakistan. The plums were washed with tap water and peeled manually. The peels were dried in hot air oven at 45°C for 72 h followed by mechanical grinding (Philips Co. Ltd., China). The powder plum peels were stored in zip lock bags covered with aluminum foil at 4°C until further use.

### 2.1. Optimization of Bioactive Compound Extraction

UAE of plum peels was optimized by RSM using the Design-Expert® software (Minneapolis, MN, USA) at a fixed frequency (20 kHz) and sample to solvent ratio (1 g/20 ml). Temperature (35, 45, and 55°C), time (15, 30, and 45 min), and ethanol concentration (50, 70, and 90%) were used as independent extraction variables. The different concentrations of ethanol were prepared by diluting ethanol with 0.1 N HCl (*v*/*v*). The powdered peels were placed in a beaker containing ethanol and subjected to ultrasonic processor (LSP-500, Industrial Sonomechanics, USA). Total phenolic content (TPC), total anthocyanin content (TAC), and DPPH radical scavenging activity were selected as the response variables.

### 2.2. Determination of TPC, TAC, and Antioxidant Activity

TPC of plum peel extract (PPE) was estimated by Folin-Ciocalteu reagent (Sigma-Aldrich, USA) as described by Sadiq et al. [17]. Gallic acid was used to develop a standard curve, and results were expressed as mg of gallic acid equivalent (GAE) per gram of sample. Antioxidant activity of PPE was evaluated by using standard DPPH inhibition assay [23].

TAC of PPE extract was estimated by pH differential method as described by Lee et al. [24] with slight modifications. PPE (1 ml) was added to 0.025 M potassium chloride buffer (9 ml, pH 1); similarly, PPE (1 ml) was added to 0.4 M sodium acetate buffer (9 ml, pH 4.5). The absorbance of resulting solutions was recorded within 20-50 min by UV-Visible spectrophotometer (Aurius 2000 series, Cecil Instruments, England) at 700 nm and 520 nm against blank. TAC was estimated by using equation (1) and expressed as mg of cyanindin-3-glucoside equivalent/g of sample.

(1) $$\begin{array}{c} \text{TAC }\left(\text{mg}/\text{g}\right)=\frac{\text{Abs}\times \text{MW}\times \text{DF}\times 1000}{\varepsilon \times L}, \end{array}$$

where absorbance (Abs) = [(*A*_520_ − *A*_700_)_pH 1.0_] − [(*A*_520_ − *A*_700_)_pH 4.5_], *ε* (cyanidin − 3 − glucoside molar absorbance) = 26,900 L mol^−1^ cm^−1^, *L* is the path length of the cell (1 cm), MW (molecular weight of anthocyanins) = 449.2 D, and DF is the dilution factor.

### 2.3. Bioactive Potential of Optimized PPE

#### 2.3.1. Antioxidant Activity

The optimized extraction conditions (49°C, 37 min, and 68% ethanol) were determined by using desirability function of Design-Expert. PPE obtained by using optimized extraction conditions was lyophilized (Christ Alpha 1-2 LDplus, Germany) and further evaluated for DPPH inhibition potential at different PPE concentrations (8000-31.25 *μ*g/ml). For antioxidant potential, IC_50_ value of PPE was determined by nonlinear regression using GraphPad Prism® version 7 (San Diego, US).

#### 2.3.2. Anticancer Activity

The anticancer activity of PPE was evaluated by using MTT cell proliferation assay and wound healing assay as described by Nelson et al. [25].

*(1) Cell Culture*. Four human cancer cell lines, DLD-1 (colon cancer), HCT-116 (colon cancer), MDA-MB-231 (breast cancer), and PC3 (prostate cancer) were cultured using DMEM cell culture medium supplemented with 10% heat inactivated fetal bovine serum and antibiotics (penicillin 100 units/ml and streptomycin 100 *μ*g/ml). All cell culture media and reagents were purchased from Gibco, Thermo Fisher Scientific, USA. Cells were maintained in cell culture flasks with filtered caps for air exchange and kept at 37°C with 5% CO_2_ in an incubator. Cells were maintained below confluence by passaging every two to three days.

*(2) MTT Cell Proliferation Assay*. MTT (methylthiazolyldiphenyl-tetrazolium bromide) cytotoxicity assay was used to assess the growth inhibition of human cancer cell lines in the presence of PPE. Vacuum dried samples of PPE were dissolved in DMEM at the final concentration of 20 mg/ml, and the solution was sterilized by passing through 0.22 *μ*m syringe filter. For the assay, 8000 DLD-1 and HCT 116 and 4000 MDA-MB-231 and PC3 cells were seeded in 96-well flat bottom cell culture plates. Cells were grown in the final volume of 100 *μ*l of complete DMEM in the presence of varying concentrations of PPE (10 *μ*g/ml to 5 mg/ml) or doxorubicin as positive control. After 72 h of incubation, 10 *μ*l of 5 mg/ml sterile solution of MTT was added in each well, and cells were further incubated for 4 h. Media was removed from wells, and 100 *μ*l of isopropanol containing 0.04 N HCl was added in each well to dissolve the formazan crystals. Assay plates were read at 492 nm, and after subtracting background absorbance, data was normalized considering untreated wells as zero percent growth inhibition. Cell survival was plotted against log of PPE concentration, and by nonlinear regression curve fitting of normalized data, IC_50_ values were calculated using the GraphPad Prism software.

*(3) Wound Healing Assay*. MDA-MB-231 and PC3 cells were seeded in 6-well cell culture plates at 1 × 10^5^ cells per well. Both cell lines were grown for a day to obtain confluent monolayers which were wounded by scratching with a sterile 200 *μ*l tip. Cells were washed twice with serum free DMEM media to remove detached cells. Scratch wounds were allowed to heal in reduced serum DMEM medium (2.5% FBS) in the presence of different concentrations of PPE. Wound gaps were imaged using inverted phase contrast microscope at 0, 12, and 24 h. Wound healing was quantified by measuring wound areas using the ImageJ software. Percentage healing was calculated by normalizing wound areas after healing to areas at the start of experiment, presented as percentage of untreated controls.

### 2.4. Antibacterial Activity

Antibacterial activity of PPE was evaluated against *Escherichia coli* (ATCC# 8739), *Staphylococcus aureus* (ATCC# 25923), and *Salmonella typhimurium* (ATCC# 14028) by agar well diffusion method [26]. Each inoculum was adjusted to 0.5 McFarland standard and applied on the surface of nutrient agar (Oxoid, UK) plate using sterilized cotton swab. With sterilized cork borer, 10 mm wells were made in each plate, and 100 *μ*l from different concentrations (100, 50, 25, and 12.5 mg/ml) of PPE was added into each well. The plates were incubated at 37°C for 24 h, and diameter of inhibition zone was recorded.

### 2.5. Food Preservation Potential of PPE

Preservation potential of PPE was evaluated in fresh grapes. Two different concentrations of PPE (25 mg/ml and 50 mg/ml) were added into 1% (*w*/*v*) chitosan (CS) solution and emulsified by the addition of glycerol (1%, *v*/*v*). The grapes were artificially inoculated with the microbial suspension of *S. aureus* (10^4^ CFU/ml) for 90 s followed by draining. The grapes were kept at room temperature for 1 h to aid bacterial adherence and dipped into CS-PPE coatings for 1 min. The grapes were kept at 25°C, and microbiological analysis was performed daily for 5 days by following Mehmood et al. [27]. At each time interval, grapes were homogenized in peptone saline solution (0.1%, *w*/*v*), and total colonies were counted and reported as CFU/g of grapes.

### 2.6. FTIR Analysis of Optimized PPE

PPE extract was analyzed by FTIR spectrometer (Agilent Technologies, USA) in absorption mode (4000-650 cm^−1^) with a resolution of 4 cm^−1^.

### 2.7. Gas Chromatography Mass Spectrometer (GCMS) Analysis

PPE extract was analyzed by GC-MS system (GC-7890A/MS-5975C, Agilent Technologies, Santa Clara, CA, USA) with HP-5 MS capillary column. Sample injection was maintained at 200°C, and helium was used as mobile phase (1 ml/min). All the data were acquired by collecting the mass spectra in the range of 50–600 a.m.u.

### 2.8. High Performance Liquid Chromatography (HPLC) Analysis

Gradient HPLC (LC-10AT, SHIMADZU, JAPAN) was used for the separation of PPE components by using shim pack CLC-ODS (C-18), 25 cm × 4.6 mm, 5 *μ*m column. Gradient mobile phase was used for separation of phenolic compounds: A (H_2_O: acetic acid, 94 : 6; pH = 2.2) and B (acetonitrile 100%) (0-15 min = 15% B, 15-30 min = 45% B, 30-45 min = 100% B). UV–Visible detector (Shimadzu, SPD-10AV) was monitored at 280 nm, and phenolics were identified by comparing retention time and UV-Visible spectra with standards [28].

### 2.9. Statistical Analysis

Tukey's HSD test was used to determine significant differences (*p* < 0.05) among treatments by using SPSS statistical software package (SPSS, version 22.0, USA).

## 3. Results and Discussion

### 3.1. Optimized Extraction of Bioactive Compound

UAE of black plum peel was optimized by Box-Behnken design, and effects of independent extraction parameters (time, temperature, and solvent concentration) on response variables (TPC, TAC, and DPPH inhibition) are summarized in Table 1. UAE of plum peel was carried by different set of extraction conditions, and TAC, TPC, and DPPH inhibitions were observed in the ranges of 1.62-6.22 mg/g, 2.23-7.38 mg GAE/g, and 52-94%, respectively. The result indicated that the optimal extraction was at 45°C, 30 min, and 70% ethanol concentration which corresponded to TAC, TPC, and DPPH inhibition values of 5.78 mg/g, 5.53 mg GAE/g, and 94%, respectively. Traore et al. [29] reported that black plum peel contained significantly high TPC (383.03 mg GAE/100 g) than the flesh of plum (202.51 mg GAE/100 g). Anthocyanin content of plum varies with the variety, maturation stage, and environmental conditions. Wang et al. [30] reported TAC of content in Myrobalan plum peel in the range of 1.93-1986 mg/g of peel, which was similar to the findings of this study.

UAE is gaining considerable interest as a sustainable, green, and suitable technique for extraction of bioactive compounds [31]. Ethanol as extraction solvent is a viable option for extraction of natural and bioactive compounds due to its low cost and environment friendly nature [32]. The dilution of ethanol with water increases the polarity of extraction solvent, and acid addition facilitates to dissolve plant membranes, resulting in efficient extraction of target components [33].

The influence of extraction parameters on response variables was evaluated by quadratic model, and the model was well fitted to the data which was confirmed by the *R*^2^ (coefficient of determination) values of 0.829, 0.887, and 0.946 for TAC, TPC, and DPPH inhibitions, respectively. Lack of fit was insignificant which indicated that data was well fitted. All the extraction parameters influence the response variables; however, the nature of influence was only significant for ethanol concentration which significantly (*p* < 0.05) influences all the response variables (Table S1). Previous studies reported an increase in TPC and antioxidant activity by increasing ethanol concentration and extraction time; however, after an optimum level, further increase in solvent concentration and extraction time was associated with decrease in the TPC and antioxidant activity of natural extracts [23, 34].

The extraction of peel sample was finally carried out at optimized extraction conditions (49°C, 37 min, and 68% ethanol), determined by using desirability function of Design-Expert, and experimental values of 5.42 ± 0.61 mg/g, 6.217 ± 0.76 mg GAE/g, and 89 ± 2.13% were obtained for TAC, TPC, and DPPH inhibitions, respectively.

### 3.2. Antioxidant Activity of PPE

**Figure 1:**
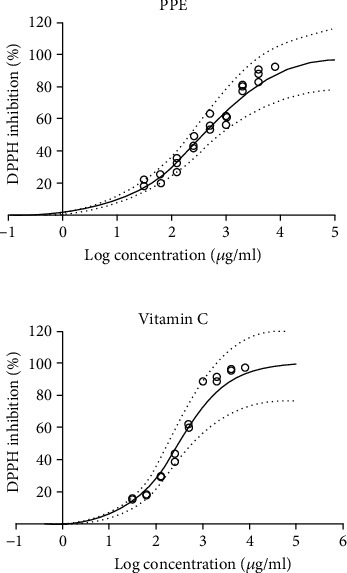
DPPH inhibition (%) of plum peel extract and vitamin C at different concentrations, using nonlinear regression.

Antioxidant activity of PPE increased with increase in concentration and at highest test concentration (8000 *μ*g/ml), 92.31 ± 0.27% DPPH inhibition was observed; however, at the same concentration, vitamin C resulted in 97.44 ± 0.21% DPPH inhibition (Figure 1 and Table S2). IC_50_ values representing the concentration of test compound required for 50% DPPH inhibition were estimated by nonlinear regression and determined as 360.6 *μ*g/ml for PPE and 298 *μ*g/ml for vitamin C. Traore et al. [29] reported that the antioxidant activity of black plum peel was almost 3 times higher than the plum flesh and IC_50_ values of plum peel for DPPH inhibition were reported in the range of 0.48-0.91 mg/ml. Antioxidant activity and various other bioactive potential of fruits and their byproducts are associated with the presence of different phenolic compounds [30]. Phenolics scavenge the stable DPPH free radical by donating their hydrogen atoms, thus reducing it into the yellow-colored diphenyl-picryl-hydrazine [35].

### 3.3. Anticancer Activity

#### 3.3.1. MTT Cell Proliferation Assay

**Figure 2:**
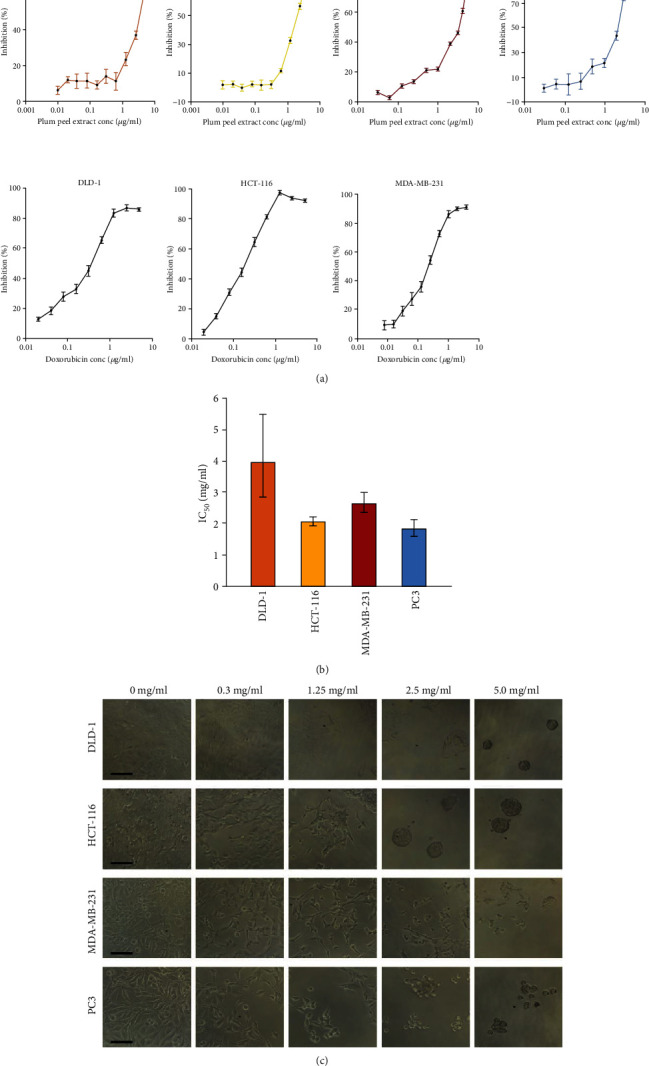
Cytotoxic effects of plum peel extract (PPE) on cancer cell lines in vitro. (a) Results of MTT cytotoxicity assay showing increase in growth inhibition of DLD-1, HCT-116, MDA-MB-231, and PC3 cells with increasing concentration of PPE. Doxorubicin was used as a positive control. Error bars show standard error of mean of four wells. (b) IC_50_ of PPE, values were obtained by nonlinear regression curve fitting of normalized cell survival; error bars represent 95% confidence interval. (c) Phase contrast microscopy, images were obtained after 72 hours of treatment with PPE; scale bar represents 100 *μ*m.

To quantify the inhibitory effect of PPE on the proliferation of DLD-1, HCT-116, MDA-MB-231, and PC3, cells were cultured in the presence of PPE, concentration ranging from 10 *μ*g/ml to 5 mg/ml. Results of in vitro MTT assay showed that PPE treatment significantly reduced cell proliferation in dose dependent manner (Figure 2(a)). All tested cell lines showed growth inhibition, and the IC_50_ values calculated were DLD-1, 3.96 mg/ml (95% CI 2.87-5.46 mg/ml); HCT-116, 2.06 mg/ml (95% CI 1.93-2.2 mg/ml); MDA-MB-231, 2.66 mg/ml (95% CI 2.36-2.99 mg/ml); and PC3, 1.85 mg/ml (95% CI 1.61-2.12 mg/ml). Doxorubicin a commonly used chemotherapeutic drug was used as positive control.

#### 3.3.2. Wound Healing Assay

**Figure 3:**
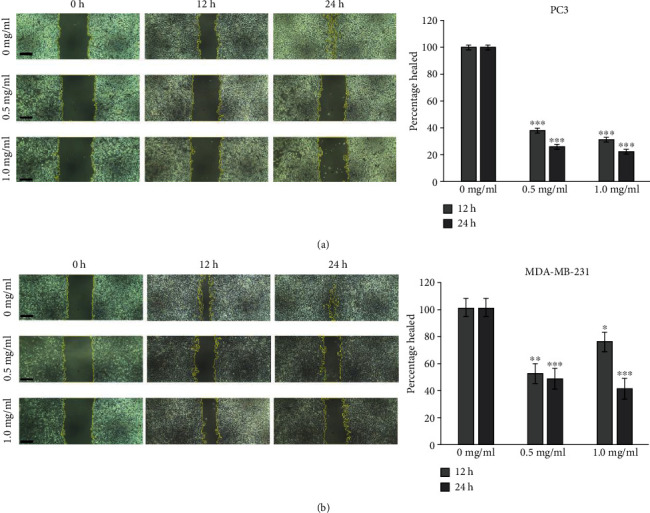
Reduction in migratory capacity of PC3 (a) and MDA-MB-231 (b) cells after PPE treatment. Left: phase contrast images of wound healing assay; cell monolayers were scratched and allowed to heal in the presence of PPE. Scratched areas were photographed at regular time intervals, yellow lines represent wound areas calculated using the ImageJ software, and scale bar represents 250 *μ*m. Right: quantitative analysis of wound healing. Percentage healing was calculated by normalizing wound areas after healing to areas at start of experiment, shown as percentage of untreated controls. Error bars represent SEM. Student's *t*-test between treated and untreated cells. ^∗^*p* < 0.05, ^∗∗^*p* < 0.005, and ^∗∗∗^*p* < 0.0005.

To quantify the impact of PPE on migration of cancer cells, we performed in vitro wound healing assay using PC3 and MDA-MB-231 cells. Results presented in Figures 3(a) and 3(b)showed statistically significant inhibition of migratory ability of cancer cells. The inhibitory effect was more noticeable for PC3 cells where 12 h of treatment with 0.5 and 1.0 mg/ml PPE resulted in reduction in their wound closure ability to 37 ± 1.7% and 31 ± 1.6% of untreated control cells, respectively. Similarly, 24 h of treatment with 0.5 and 1.0 mg/ml PPE reduced the healing ability to 25 ± 1.6% and 22 ± 1.0% of control cells, respectively. For breast cancer MDA-MB-231 cells, 24 h of incubation with 0.5 and 1.0 mg/ml PPE reduced the healing ability to 48 ± 7.3% and 41 ± 7.8% of untreated control.

Previous studies reported that plum extracts exhibited the ability to decrease the viability and proliferation of hepatocellular and colon cancer cell lines, and at a high concentration of plum extract (2 mg/ml), 87% of grown inhibition was observed ([36, 37]). Plum extract was reported to induce the cleaved caspase-8 protein expression which is responsible for inducing cancer cell apoptosis [38]. Plum extract was also reported to inhibit the cancer cell migration [39].

### 3.4. Antibacterial Activity and Food Preservation Potential of PPE

Antibacterial activity of PPE was increased in concentration-dependent manner, and at highest test concentration (100 mg/ml), inhibition zones of 25 ± 1, 21.33 ± 1.52, and 25.33 ± 0.57 mm were observed against *E. coli*, *S. typhimurium*, and *S. aureus*, respectively (Table 2).

PPE + CS-based coating restricted the growth of *S. aureus* in artificially inoculated grapes. After 5 days of storage, the bacterial count was 3.54 ± 0.04 and 4.86 ± 0.04 log CFU/g in grapes treated with CS + PPE (50 mg/ml) and CS alone, respectively (Table 3). The grapes treated with the combination of CS and PPE remained below 4 log CFU/g, which is maximum allowable limit recommended by Food and Drug Administration (FDA) for fruits. However, control treatments (grapes without PPE) were found exceeding 4 log CFU/g after 1 day of storage.

Plums are rich in phenolic compounds which are associated with various bioactive potentials including antibacterial effect. Freeze dried plum powder extract was reported to inhibit the growth of various Gram positive and Gram negative bacteria [40]. Valtierra-Rodríguez et al. [41] reported that the plum fruit extract restricted the growth of *Campylobacter jejuni* and *Campylobacter coli* in poultry skin inoculated with these pathogens. Plum products have been also effective in inhibiting the growth of *Salmonella* and *E. coli* O157:H7 [42]. Nair et al. [43] used the pomegranate peel extract with CS and alginate to preserve. They found the guava fruit and found that peel extract in combination with CS was very effective in extending the shelf life of the fruit.

### 3.5. FTIR Analysis

**Figure 4:**
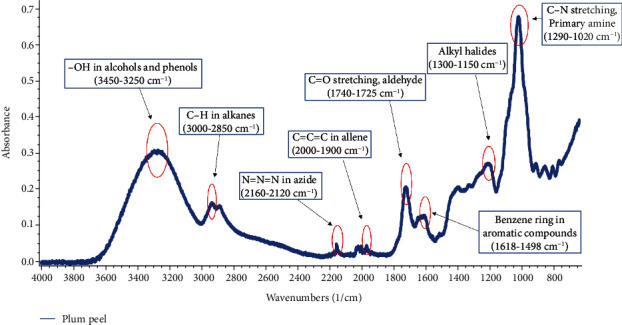
FTIR spectrum of plum peel extract.

The characteristic functional groups were assigned to corresponding peaks by following D'Angelo and Zodrow [44]. The assignment of functional group to corresponding functional groups is presented in Figure 4 and Table S3. The intense peak in the range of 1290-1020 cm^−1^ was attributed to C-N stretching of primary amines. The peak in the range of 3450–3250 cm^−1^ assigned to -OH functional group in alcohols and phenols [45]. Peaks in the range of 1300–1150 cm^−1^, 1740-1725 cm^−1^, 3000–2850 cm^−1^, 1618–1498 cm^−1^, and 2160-2120 cm^−1^ were assigned to alkyl halide, C=O stretching, C-H in alkanes, benzene ring in aromatic compounds, and N=N=N in azide, respectively.

### 3.6. GCMS and HPLC Analyses of PPE

The major phytoconstituents identified in PPE were 2-cyclopenten-1-one, 2-hydroxy-, 2,4-dihydroxy-2,5-dimethyl-3(2H)-furan-3-one, 2H-pyran-2, 6(3H)-dione, butanoic acid, 3-methylbutyl ester, 4H-pyran-4-one, 2,3-dihydro-3,5-dihydroxy-6-methyl, 5-hydroxymethylfurfural, tri(1, 2-propyleneglycol), monomethyl ether, n-hexadecanoic acid, 9,12-octadecadienoic acid, methyl ester, and 9,9-dimethoxybicyclo[3.3.1]nona-2,4-dione (Table 4 and Figure S1). Plums were reported to exhibit varieties of volatile compounds such as esters, ethers, ketones, alcohols, and lactones [46]. 2-Cyclopenten-1-one, 2-hydroxy-, 2,4-dihydroxy-2,5-dimethyl-3(2H)-furan-3-one, and 2H-pyran-2,6(3H)-dione were reported to exhibit antioxidant potential, whereas 4H-pyran-4-one, 2,3-dihydro-3,5-dihydroxy-6-methyl was found to be associated with antipathogenic potential [47].

HPLC was used for the identification of bioactive phenolic compounds in PPE (Table 5). The major phenolics detected were ferulic acid (47.87 mg/kg), sinapic acid (9.15 mg/kg), quercetin (7.44 mg/kg), gallic acid (3.24 mg/kg), m-coumaric acid (2.59 mg/kg), and vanillic acid (1.12 mg/kg). Fanning et al. [13] identified phenolic compounds in different cultivars of Japanese plum and reported that quercetin was one of the dominating phenolic compound (2-27 mg/100 g) present in different varieties of plum. Ferulic acid, coumaric acid, and quercetin have been reported to exhibit strong antioxidant and anticancer activities [14, 48].

## 4. Conclusion

Natural food preservatives have been associated with various health benefits; however, high cost hinders their industrial applications. In this study, bioactive compounds with antibacterial, antioxidant, and anticancer potential were extracted from plum peels by optimizing UAE. Phytochemical analysis revealed the presence of high phenolic and anthocyanin contents in PPE. Optimized PPE was found rich in secondary metabolites which exhibit remarkable antibacterial, anticancer, and antioxidant potential. Due to the presence of high phenolic and anthocyanin content, PPE can be used in the development of functional foods, natural active ingredient-based pharmaceutical, and cosmetic products.

## Figures and Tables

**Table 1:** Optimization of extraction of bioactive compounds from black plum peels.

Exp. no.	Independent variables			Response variables		
	Temperature (°C)	Time (min)	Solvent (%)	TAC (mg/g)	TPC (mg GAE/g)	DPPH inhibition (%)
1	35	30	50	4.69	5.88	73.03
2	55	30	90	3.94	2.48	84.92
3	45	45	90	3.51	2.41	89.31
4	45	30	70	5.78	5.53	94.0
5	45	15	90	3.27	3.79	70.86
6	45	30	70	4.94	6.01	93.06
7	45	15	50	1.62	3.57	86.41
8	35	30	90	5.03	2.23	84.58
9	35	15	70	4.13	2.67	91.71
10	55	15	70	5.37	5.90	92.62
11	55	45	70	4.89	6.30	93.0
12	45	30	70	5.27	6.15	84.68
13	45	45	50	6.22	7.38	52.59
14	55	30	50	5.76	6.092	63.98
15	35	45	70	4.94	4.375	88.74
16	45	30	70	2.05	6.63	88.44
17	45	30	70	4.19	5.025	91.98

**Table 2:** Antibacterial activity of optimized plum peel extract.

Extract conc.	Diameter of zone of inhibition against pathogens (mm)		
mg/ml	*Escherichia coli*	*Salmonella typhimurium*	*Staphylococcus aureus*
100	25 ± 1^a^	21.33 ± 1.52^a^	25.33 ± 0.57^a^
50	22.66 ± 0.57^b^	21.33 ± 2.08^a^	23.66 ± 1.52^a^
25	21.33 ± 0.57^b^	19.66 ± 1.15^a^	20.33 ± 0.57^b^

Different superscript small letters indicate means which are significantly (*p* < 0.05) different.

**Table 3:** Microbiological analysis of fresh grapes treated with PPE-based coating.

Days	Chitosan (CS) (log CFU/g)	Chitosan+ PPE 25 mg/ml (log CFU/g)	Chitosan +PPE 50 mg/ml (log CFU/g)	Control (log CFU/g)
1	3.04 ± 0.09^a^	2.64 ± 0.18^a^	2.49 ± 0.19^a^	4.86 ± 0.04^a^
2	3.19 ± 0.05^ab^	3.01 ± 0.06^b^	2.86 ± 0.08^b^	5.15 ± 0.03^b^
3	3.35 ± 0.08^bc^	3.18 ± 0.04^bc^	3.06 ± 0.09^bc^	6 ± 0.08^c^
4	3.48 ± 0.05^c^	3.34 ± 0.05^c^	3.25 ± 0.1^c^	6.74 ± 0.07^d^
5	4.86 ± 0.04^d^	3.63 ± 0.07^d^	3.54 ± 0.04^d^	7.35 ± 0.05^e^

Different superscript small letters indicate means which are significantly (*p* < 0.05) different.

**Table 4:** GCMS analysis of plum peel extract.

Serial #	Retention time	% of total	Compounds	Molecular formula
1	7.066	26.027	2-Cyclopenten-1-one, 2-hydroxy-	C_5_H_6_O_2_
2	7.833	2.240	2,4-Dihydroxy-2,5-dimethyl-3(2H)-furan-3-one	C_6_H_8_O_4_
3	8.123	4.782	2H-Pyran-2,6(3H)-dione	C_5_H_4_O_3_
4	8.939	29.882	Butanoic acid, 3-methylbutyl ester	C_9_H_18_O_2_
5	10.588	5.703	4H-Pyran-4-one, 2,3-dihydro-3,5-dihydroxy-6-methyl	C_6_H_8_O_4_
6	11.881	19.261	5-Hydroxymethylfurfural	C_6_H_6_O_3_
7	13.168	1.959	Tri(1, 2-propyleneglycol), monomethyl ether	C_10_H_22_O_4_
8	20.364	4.027	n-Hexadecanoic acid	C_16_H_32_O_2_
9	22.013	4.355	9,12-Octadecadienoic acid, methyl ester	C_19_H_34_O_2_
10	22.237	1.765	9,9-Dimethoxybicyclo[3.3.1]nona-2,4-dione	C_11_H_16_O_4_

**Table 5:** HPLC analysis of plum peel extract.

Compound name	Retention time	Area (%)	Concentration (mg/kg)
Quercetin	2.773	2.4	7.44
Gallic acid	4.937	1.5	3.24
Vanillic acid	13.267	0.3	1.12
m-Coumaric acid	20.140	3.6	2.59
Ferulic acid	21.587	11.2	47.87
Sinapic acid	26.647	7.6	9.15

## Data Availability

All the data has been incorporated within the study and supplementary file.

## Supplementary Materials

Supplementary Materials Table S1: analysis of variance table for total anthocyanin content, total phenolic content, and antioxidant activity with 95% confidence level. Table S2: %age DPPH inhibition of optimized PPE extract and vitamin C. Table S3: assignment of FTIR peaks to the functional groups in the PPE. Figure S1: total ion chromatogram of plum peel extract.
