# Supplementary material for: Determination of Phenolic Compounds and Bioactive Potential of Plum (Prunus salicina) Peel Extract Obtained by Ultrasound-Assisted Extraction
Source: Biomed Res Int. 2022 Aug 2;2022:7787958. doi: 10.1155/2022/7787958 (PMC9433295; doi:10.1155/2022/7787958)
Supplement: Supplementary Materials — Table S1: analysis of variance table for total anthocyanin content, total phenolic content, and antioxidant activity with 95% confidence level. Table S2: %age DPPH inhibition of optimized PPE extract and vitamin C. Table S3: assignment of FTIR peaks to the functional groups in the PPE. Figure S1: total ion chromatogram of plum peel extract. [file 7787958.f1.docx]

**Supplementary File**

**Determination of phenolic compounds and bioactive potential of plum (*Prunus salicina*) peel extract obtained by ultrasound assisted extraction**

**Table S1.** Analysis of variance table for total anthocyanins content, total phenolic content and antioxidant activity with 95% confidence level

| Source | TAC (mg/g) | | TPC (mg GAE/g) | | DPPH (%) | |
| --- | --- | --- | --- | --- | --- | --- |
|  | **F**  **Value** | **p-value**  **Prob > F** | **F**  **Value** | **p-value**  **Prob > F** | **F**  **Value** | **p-value**  **Prob > F** |
| Model | 3.78 | 0.0468* | 6.15 | 0.0129* | 13.64 | 0.0012* |
| A-Temperature | 0.87 | 0.3812 | 5.49 | 0.0517 | 0.092 | 0.7709 |
| B-Time | 1.51 | 0.2584 | 3.54 | 0.1018 | 2.36 | 0.1684 |
| C-Solvent Conc | 12.58 | 0.0094* | 24.91 | 0.0016* | 21.07 | 0.0025* |
| AB | 0.69 | 0.4337 | 0.59 | 0.4681 | 0.16 | 0.6974 |
| AC | 0.28 | 0.6113 | 7.312E-004 | 0.9792 | 1.29 | 0.2934 |
| BC | 9.39 | 0.0182 | 9.34 | 0.0184 | 39.98 | 0.0004* |
| A^2^ | 0.50 | 0.5012 | 2.00 | 0.2003 | 0.53 | 0.4920 |
| B^2^ | 0.20 | 0.6681 | 1.28 | 0.2949 | 0.035 | 0.8577 |
| C^2^ | 7.53 | 0.0287* | 7.20 | 0.0314* | 57.42 | 0.0001* |
| Lack of Fit | 1.46 | 0.3520 | 3.15 | 0.1485 | 1.37 | 0.3734 |

**Table S2.** %age DPPH inhibition of optimized PPE extract and vitamin C

| Quantity  (µg/ml) | Vitamin C | Black Plum Peel Extract |
| --- | --- | --- |
| 8000 | 97.44 ± 0.21ᵃ | 92.31 ± 0.27ᵃ |
| 4000 | 95.97 ± 0.85ᵃ | 87.35 ± 3.91ᵃᵇ |
| 2000 | 89.50 ± 1.74ᵇ | 79.47 ± 1.82ᵃᵇ |
| 1000 | 88.14 ± 0.41ᵇ | 59.59 ± 3.05ᵇ |
| 500 | 60.26 ± 1.50ᶜ | 57.45 ± 5.35ᶜ |
| 250 | 41.73 ± 2.80ᵈ | 44.81 ± 3.95ᶜ |
| 125 | 29.63 ± 1.09ᵉ | 31.84 ± 4.32ᵈ |
| 62.5 | 18 ± 1ᶠ | 21.80 ± 3.13ᵉ |
| 31 | 15.87 ± 1.02ᶠ | 19.77 ± 2.45ᶠ |

*Different superscripts small letters indicate means which are significantly (p<0.05) different

**Table S3.** Assignment of FTIR peaks to the functional groups in the PPE

| Range (cm^₋1^) | Group and Class of Compound | PPE  (cm^₋1^) |
| --- | --- | --- |
| 3450–3250 | OH  In alcohols and phenols | 3298.2 |
| 3000–2850 | C-H  In alkanes | 2937.6 |
| 2160-2120 | N=N=N  In azide | 2160.8 |
| 2000-1900 | C=C=C  In allene | 1974.7 |
| 1740-1725 | C=O stretching  Aldehyde | 1729.4 |
| 1618–1498 | Benzene ring  in aromatic compounds | 1618.7 |
| 1300–1150 | Alkyl halides | 1215.8 |
| 1290-1020 | C-N stretching  Primary amine | 1029.3 |

**Figure S1.** Total ion chromatogram of plum peel extract
